# Buffalo Medical College

**Published:** 1853-11

**Authors:** 


### EDITORIAL DEPARTMENT.

*Buffalo Medical College.*—The dissolution of the Medical College at Geneva, has left the Buffalo Medical College the sole representative of the interests of medical education in western New York. Of the causes which have brought about the decay, and final downfall, of an institution so honorable and useful as that at Geneva, we have nothing to say. But we feel confident that in speaking to the profession of this, (the wealthiest and most prosperous of all sections of the country,) of the interests and prospects of their only medical institution, we shall only re-echo the feelings of all honorable physicians.

The medical department of the University of Buffalo is as yet a new institution. Its founders based their calculations of ultimate success, upon the growing greatness of the city of Buffalo, its intimate connection by traveling facilities with an extended area of country, its facilities for clinical instruction and dissections, and, finally, and chiefly, upon the individual energies and capacities of its teachers. Its organization was hailed by the profession as a good omen of professional advancement; and it has from its inception been sustained by the unfaltering hands of steadfast friends. Already it has a large number of alumni scattered over the country, whose affections still remain with their *alma mater*, and whose interest in its welfare is unchanging.

As we remarked before, the dissolution of the Geneva school has left to Buffalo the honor and grave responsibility of sustaining alone the cause of medical education in western New York. We offer, then, no apology for presenting its claims to the profession.

Its location is one eminently favorable. It is situated at a great center of communication, to be soon rendered still more accessible and central, by the extension of railroad communication to Pittsburg and to Canada. It possesses those advantages most essential to the prosperity of any school: a location in a large and growing city, and unusual facilities for clinical instruction. The city of Buffalo is now assuming a metropolitan importance. Its size and population are characterized by a growth rapid and healthy, the latter now estimated to number 80,000 souls within the present extended city limits. But the single fact of location in a large city, is not in itself valuable, unless the advantages incident thereto are made available for the purposes of instruction.

Early in the history of the school, its friends took measures to secure the erection of a hospital, and the perpetual use of its wards for clinical teaching. This movement, which was in itself one of the highest beneficence, would probably have been successfully carried through were it not for an opposition which, we regret for the honor of our city to say, originated in the ranks of a disaffected and jealous portion of the profession. But another hospital, equally advantageous to the school, was erected and endowed, and it now exists under the direction of the Sisters of Charity, in a state of completeness and prosperity which affords the warmest satisfaction to its friends. To illustrate the advantages which it affords to the college, it is only necessary to state that it has nearly as many beds as has the Pennsylvania Hospital, (the only one open to medical students in Philadelphia,) and that, consequently, the same amount of hospital instruction is here provided for the students of a single school, as is supplied to the twelve hundred students annually congregated in Philadelphia. Its immediate proximity to the college edifice, and the fact that the teachers in the school are also the attending physicians of the hospital, and are thus able to refer fully to the history of any individual case, are no trifling advantages. Lastly, in this connection, we must not omit the fact that the Buffalo school, (standing alone in this respect,) makes attendance on the cliniques compulsory. All students matriculating at the college are admitted by the act of matriculation, and without extra charge, to the advantages of the hospital. No other school has as yet made such an arrangement; and it is impossible that it can be very widely imitated, as few other schools have it in their power so to do.

Such are the material advantages of the Buffalo Medical College. Of its teachers we do not propose to speak in detail. Our connection with them is one too intimate and friendly, to render our remarks on this score entirely unprejudiced, and we therefore confine ourselves to the statement of facts relative to its collateral advantages. One thing, however, we may say: circumstances honorable in themselves, and flattering to those members of the faculty concerned, have occasioned some changes in the ranks of its corps of professors. A peculiar fondness for its teachers seems to have actuated certain wealthy and popular schools, to supply vacancies in their own ranks, from the chairs at Buffalo. Of this, however we may regret it, we cannot complain. But the institution is now organized on so permanent a basis, and its faculty is now so made up, that it is probable that few or no changes will occur hereafter. It has been the aim of the council in filling the vacancies which have occurred, to supply them with men who would not only bring talent and energy, but an individual interest to the support of the school.

To this end Prof. Moore has resigned those chairs in eastern schools which he has so long and ably filled, and now confines his whole strength to the interests of Buffalo.

We have spoken thus freely and fully of the school for several reasons. Our own connection with it is not such as to imply any personal motive or vanity in penning this article; we felt that the institution had unusual merits and advantages; and, finally, we knew our audience, and have spoken as to friends, who feel with us a deep interest in the cause of medical education, and whom we believe join us in wishing continued and increased prosperity to the school. Before this will have reached our readers, the annual course of lectures will have commenced, and we shall take pleasure in informing them of its progress and prospects. S. B. H.

